# Analyzing the Use of Accelerometers as a Method of Early Diagnosis of Alterations in Balance in Elderly People: A Systematic Review

**DOI:** 10.3390/s19183883

**Published:** 2019-09-09

**Authors:** Raquel Leirós-Rodríguez, Jose L. García-Soidán, Vicente Romo-Pérez

**Affiliations:** 1 Faculty of Physical Therapy, University of Vigo; Campus a Xunqueira, s/n, 36156 Pontevedra, Spain; 2 Faculty of Education and Sport Sciences, University of Vigo; Campus a Xunqueira, s/n, 36156 Pontevedra, Spain

**Keywords:** wearables, kinematics, sensors, motion analysis, postural balance

## Abstract

Alterations of balance are a growing public health problem as they affect one in three adults over the age of 65, and one in two over the age of 80. Identifying the factors that affect postural stability is essential in designing specific interventions to maintain the independence and mobility of older people. The aim of this review was to understand the use of accelerometers in order to assess the balance in older people. Analyzing the most appropriate evaluation methodology and protocolizing it will optimize the processes of early identification of balance alterations. However, quantitative assessment methods of balance are usually limited to a laboratory environment, a factor that can be overcome by accelerometers. A systematic search was carried out across eight databases where accelerometers were employed to assess balance in older people. Articles were excluded if they focused on sensor design and did not measure balance or apply the technology on targeted participants. A total of 19 articles were included for full-text analysis, where participants took part in the balance evaluation monitored by accelerometers. The analysis of spatio-temporal parameters and the magnitude of the accelerations recorded by the devices were the most common study variables. Accelerometer usage has potential to positively influence interventions based on physical exercise to improve balance and prevent falls in older people.

## 1. Introduction

### 1.1. Background

Balance and, consequently, the control of position is a complex skill based on the interaction of sensorimotor processes, whose main objective is the maintenance by the individual of the orientation of the desired position that resists in equilibrium the force of the gravity acting on the body [1]. This is done through the integration of somato-sensory information, which includes the coordinated and integrated function of proprioceptive, visual, and vestibular information [2,3]. The maintenance of postural balance involves the coordination of motor strategies to stabilize the center of mass (CM) during movements made by the individual and/or external stability disturbances [4,5]. To facilitate the incorporation of older adults into programs to improve balance and evaluate the effectiveness of such programs, either with caution or for the treatment of instability, early diagnosis methods are needed; that is, methods that are sensitive to small changes in the functioning of postural control systems. However, the multifactor nature of balance largely prevents the quantitative, global, and reliable assessment of this ability in clinical practice.

### 1.2. Balance Evaluation

The deterioration of balance in humans has usually been measured by valid and reliable functional and static tests [6,7]. However, the skills they analyse are particularly easy and it is common to see how people get very high scores in general. Therefore, only significant alterations are found when the deterioration of postural control is already very large [8,9,10]. This means that in the interventions there is no reliable procedure that allows for the detection of early changes in balance caused by age, nor the measurement of the variations produced by rehabilitation programs designed for different pathologies. Therefore, it is necessary to design new, more accurate and reliable diagnostic methods that allow us to detect small changes in peoples’ balance at an early period.

The multifactorial nature of equilibrium that was developed earlier made it difficult to assess deterioration in the same way. This led to the development of a wide array of tests and batteries with different approaches to the applicability of daily clinical practice. In general, an aspect common to all of them is that the resulting score depends on qualitative evaluation [11].

In the laboratory, more sophisticated instruments have been developed as tools for evaluating balance. For the study of static or posturographic balance, different evaluation instruments have been used. Posturography is divided into static, when the position is studied in normal conditions, and dynamic, when the position is studied in response to a perturbation applied to the individual or to the surface of support in which the individual is located [12]. Two of the most common instruments used for these investigations are computerized dynamic posturography and force platforms for the determination of the individual’s center of pressure (CP), an indicator of great clinical validity to identify relatively premature sensory-motor deficits [13].

The postural control analyses derived from the CP measurements provided by force platforms are usually limited to the statistical analysis of a few measurements (contributing to the typical deviation, length of the route performed by the CP, and/or the speed of movement thereof). Force platforms allow postural evaluation in different equilibrium conditions—such as standing or on a single leg, having eyes open or closed, performing cognitive tasks at the same time—which means that this tool has been the methodological instrument chosen for many research studies [14]. Therefore, this method has been considered the gold standard for the quantification of the state of postural control in relation to the maintenance of static balance, and most of the tests and clinical evaluation tests explained in the previous pages were compared to the force platform, with the objective of its validation [11,15].

Dynamic posturography consists in measuring the oscillations in postural control over an area that is, to a greater or lesser extent, unstable. This external instability can be permanent if the surface in contact with the ground is not flat, as in oscillating platforms, or caused by a sudden alteration of the support surface (such as an inclination or rotation thereof). On the other hand, instability can also be internally (or voluntarily) caused by movements such as functional reach tests (leaning forward), raising arms, and sit-to-stand, among other types [16].

Computerized dynamic posturography uses a force platform in combination with different stimuli. One model of computerized dynamic posturography widely used in research is the EquiTest (NeuroCom International, Clackamas, OR, USA), which is made up of an evaluation surface equipped with two independent force platforms (one for each foot) in the mid-lateral axis (allowing for movements of flex-extension of the ankle). It also has a front screen that allows changes to the vision conditions [17,18,19]. Computerized posturography does not provide information about the type of difficulty that the individual has in maintaining control of the position, but only quantifies the degree of functional limitation to stay in balance; this allows the prediction of the risk of falling and the evaluation of rehabilitation programs in relation to three subsystems (vestibular, sensory–sensory and visual). Other platforms have been developed from piezoelectric systems, based on the same evaluation methodology, but the mechanism emits small electrical charges as a result of the pressure exerted; that is to say, the nature of the transducers used is different [20].

In the same line, stabilizers formed by two plates with pressure sensors were developed. The bottom plate is fixed and the top allows tilting movements of up to 20 degrees in the three axes of movement. The pressure data is transmitted to a computer where the CP changes obtained during the test are recorded. Among the study variables that a stabilizer provides, in addition to those derived from the movements of the CP and its speed, is the oscillation area (area or radius of the circle that contains at least 95% of the CP points during the test). This tool, like computerized dynamic posturography, makes it possible to carry out tests of the dynamic control of the CP in which, through a screen, the patient is asked to move the CP to a specific point. This type of test, in addition to evaluations, can also be a part of the rehabilitation treatment and balance training [21,22].

These evaluation methods are theoretically in conflict with the concept of balance that was previously defined, such as the coordination of motor strategies to stabilize the CM during the movements carried out by the individual and/or external disturbances [4,5]. However, to quantitatively measure one’s balance in laboratory environments, the conventional method is to use posturography by comparing the CP scroll between the feet. The CP is the point where the vertical force vector is placed on the ground, and represents a weighted average of all the pressure on the ground support surface. It is, therefore, a parameter independent of the CM—when erect on a single foot, the CP is inside the supported foot; when erect on both feet, the CP is at a point between both feet. That is, we are talking about a parameter that is strongly conditioned by the intrinsic activity of the ankle muscles [23]. It has been shown that the variables derived from the study of the CP are highly correlated with the behavior of the CM, as the reaction force of the ground is proportional to the acceleration of the CM in static positions [24,25].

### 1.3. Accelerometers as a Clinical Evaluation Tool

The accelerometers are a type of mobile and light inertial sensor that provides a less expensive and instrumental evaluation method than those commonly used to make posturographies. An accelerometer consists of a suspended mobile bar on micro-machined springs, which provide resistance to the movement (and acceleration) of the bar. When this bar deviates, the springs perceive their acceleration. A device can hold up to three bars with their respective springs that, independently, read the movements in a dimension, providing the activity logs on the three spatial axes—these being triaxial accelerometers [26].

The unit of measurement used by these devices is the gravity unit (g), which is based on the acceleration that gravity produces on all objects in ideal conditions (without resistance or friction of any kind). An acceleration of 1 g is considered equal to the standard gravity that is 9.8 meters per second squared (m/s^2^). This is the unit of measurement for acceleration in the International System of Units [27].

Originally, the use of accelerometers was limited to the monitoring of physical activity and the measurement of the time of permanence in different activities classified according to the intensity level of the exercise or resting position (standing, sitting, or lying down). It has even been observed that these devices offer older people an incentive to practice physical activity [28]. The use of accelerometers has allowed the movements of the daily life of the individual to be quantified thanks to their portability by means of an adjustable fastening belt to the trunk, arms, or any other desired anatomical location. This aspect is of great interest in fields such as medicine, physiotherapy, and sports training, as this flexibility in the choice of the point of registration makes it a tool that can be adapted to a multitude of measurement objectives [29,30].

Accelerometers transfer the recorded data wirelessly and have the necessary potential to overcome the major drawbacks of cost, size, and installation of instrumentalized tests of balance, as well as allowing the objective and sensitive assessment of postural control in clinical and outpatient settings [31,32,33].

From the clinical point of view, it is important to know the accelerations in the three axes of space in both static and dynamic equilibrium. In fact, many studies have indicated the spatio-temporal parameters of the gait as predictors of the state of equilibrium and the risk of falling in elderly people [34,35,36]. The advantages of this technique are the lower cost of equipment, and the portability and sensitivity of the different assessment tests in static balance [37]. In addition, analysis of the progress with this technique allows us to evaluate the functional capacity and the risk of falling [38,39,40]. Therefore, accelerometers have the necessary characteristics to be able to replace the conventional force platforms to evaluate static balance [41,42]. In addition, with this method of evaluation, the study variable is the behavior of CM in space, approaching the object of evaluating the theoretical conception of the maintenance of balance [43,44,45,46,47].

Falls cause moderate to severe injuries in 30% of cases, which, for the elderly, involves fractures, functional disability, reduced levels of physical activity, and premature entry into residential care institutions. This phenomenon requires the dissemination of the pathophysiology underlying this problem and the awareness of health professionals for their proper treatment and, above all, for their early detection, which is the key to preventing falls [1,2,3]. The aim of this review was to understand the use of accelerometers in order to assess balance in older people. Determining the most appropriate evaluation methodology and protocolizing it will optimize the processes of early identification of balance alterations.

## 2. Materials and Methods

### 2.1. Search Strategy

The following databases were used to carry out a systematic search throughout the month of May 2019: Scopus, Medline, SpringerLink, IEEE Xplore, Web of Science (Core Collection), SPORTDiscus, ScienceDirect, and PLOS One. The search terms were ‘accelerometry/accelerometer’, ‘elderly /older adults’, and ‘balance’, and the operators for the search we used were ‘(accelerometry OR accelerometer) AND balance AND (elderly OR older adults)’. We also use the RefWorks program (ProQuest, Ann Arbor, MI, USA) to do more specific searches and also to filter the articles we have found.

### 2.2. Eligibility Criteria

Articles were included if they were published in English, published in the last five years (from 2014 to the present), included kinematics and kinetics as obtained from accelerometers, and the sample consisted of adults and/or older adults. Articles were excluded if they were a review or case study, were a conference abstract (except peer-reviewed abstracts), used wearable technology only to quantify physical activity, described a potential technology not validated/used with human subjects, or they focused on sensor design and did not measure balance or apply the accelerometry on targeted participants.

### 2.3. Selection Process

We removed from our search those articles that appeared as duplicates in the different databases we used. We also reviewed the abstracts and titles of the articles in submitting them to the exclusion and inclusion criteria. Finally, the full texts of the missing articles were located to also submit them to the inclusion criteria.

### 2.4. Data Extraction

The procedure used to obtain the data was adapted from an article by Papi et al. [48], in which they investigated the use of portable technology to measure kinematic variables of the spine. The following details were extracted from each study: objective, type of population (healthy, type of pathology), sample size, demographic data of the participants (for example, type of population, age, sex, mass, height), completed tasks, accelerometry system used, data acquisition/sampling, configuration of the participant (for example, sensor location, body fixation technique), data analysis (filters used for signal collection), kinematic and kinetic records collected from acceleration signals (performance guides), statistical research methods, and validity/reliability.

### 2.5. Quality Appraisal

The review article by Papi et al. [48] was used as a reference to create a quality control list of the articles we found. In it, we included 17 items, whose rating ranged from 0 to 2 (where 0 = no, 1 = limited, 2 = very detailed): objectives, study population and design, eligibility criteria, sampling size and methodology, adequate description of the procedure for its replication, equipment design, sensors locations, sensor attachment method, signal/data described, main outcomes and calculations, system compared to gold standard, reliability of the equipment, main findings, statistical tests, and limitations.

## 3. Results

**Figure 1:**
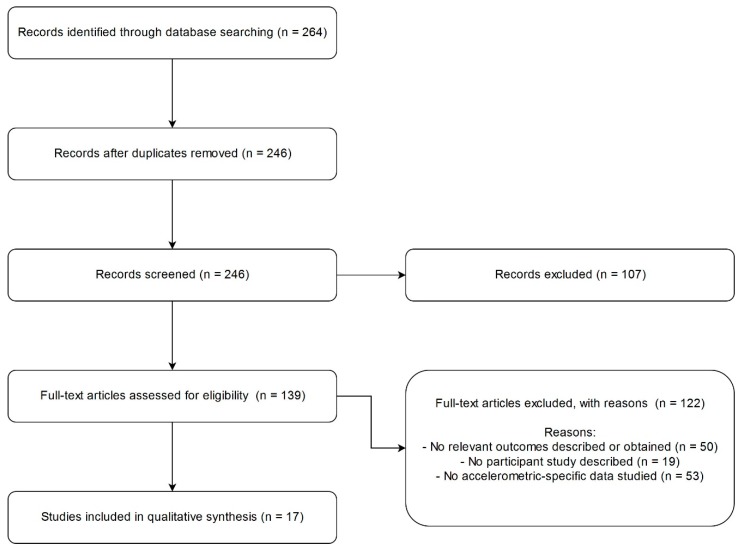
PRISMA chart detailing the article selection process.

After applying the checklist, we found 264 articles. First, we eliminated the duplicate articles, and in this way we obtained 246 articles, to which we applied the inclusion and exclusion criteria discussed previously in Section 2.2. Finally, we obtained 32 articles in which to check their possible final choice. Nineteen articles satisfied the inclusion criteria. The selection process and reasons for exclusion are presented in Figure 1.

### 3.1. Article Quality

The quality of the included papers was rated according to the following scale: low (score < 33.3%), medium (33.4–66.7%), and high (score > 66.8%) [48]. Of the articles, 1 was deemed to be of low quality [49], 14 of medium quality [50,51,52,53,54,55,56,57,58,59,60,61,62,63], and 2 of high quality [64,65]. The results from this assessment are detailed in Appendix A, Table A1, Table A2 and Table A3. None of the articles described a sampling methodology or justified the sample size. The sample size was described in all the articles and presented a variation of 12 [54] to 122 [58] in the selected articles, presenting 45 participants as an average value. Seven studies [56,57,58,60,62,63,65] had a sample equal to or greater than the average. We should point out that no article described the method and protocol used in enough sufficient detail to allow its precise replication. Two articles [49,53] did not include the detailed description of the location of the sensors used, and six articles [50,51,55,57,58,64] did.

Only seven of the articles [51,53,54,55,61,62,65] compared their measurements with a standard gold measure when testing. In three articles [58,60,63], the authors contrasted the results of the data obtained with values established in previous works, as a method of validation of the data obtained by the sensor. In Appendix A, Table A4, Table A5, Table A6, Table A7 and Table A8, the methodological aspects of each of the pieces of research analyzed are shown.

### 3.2. Types of Measuring Systems Used, Data Sampling, and Tests Performed

Commercial triaxial accelerometers were the most common measuring instrument in the studies analyzed. However, there were studies that used a mobile phone [53] and a tablet [63,65] endowed with an accelerometer and gyroscope, seeking to evaluate their validity as an instrument for the evaluation of balance. In these aforementioned studies, the results indicated that a smartphone can give a valid measure of postural stability and is able to distinguish the stratification of the risk of falls in older adults. Additionally, with the use of the tablet, the results showed that trunk accelerations in the antero-posterior and medio-lateral planes during running and different static balance positions were measured accurately. Healthy young adults, middle-aged, and older people under different sensory conditions for support and dual cognitive tasks, with the parameters of walking and balance derived from the acceleration signals proven to be valid and reliable.

The configuration of the devices was less than 55 Hz in six of the articles analyzed [50,51,52,58,59,64]. In the other studies analyzed, the measurement frequency was around 100 Hz, up to the highest measurement recorded, which was 128 Hz in the investigation by Aziz et al. [54]. However, this important methodological aspect does not appear to be described in seven of the investigations analyzed [49,53,55,57,60,62,63].

In the literature analyzed, the most common practice was to use the last lumbar vertebrae and pelvis as registration points: L3 [51,52,55,59,62,63,64], L5 [61], and the sacrum [65]. However, there were studies that used several checkpoints, such as Tung et al. [50], who analyzed the accelerations from the middle axillary line, the left iliac crest, and the ankles, and compared the reliability of the results using all the records or different combinations thereof. They found that, in general, the reduction in the number of sensors resulted in a decrease in precision that ranged from 91.1% (three sensors), 73.3–84.4% (two sensors), to 64.4‒70.0% (one sensor). Between the two combinations of sensors, the combination of left and right ankle (73.3%) produced less precise results than the combination of hip and ankle sensors (hip and left ankle, 84.4%; hip and right ankle, 82.2%). The study by Aziz et al. [54] also evaluated how to register the malleolus of the ankles, the waist, and the sternum. They observed that in order to reduce the measurement points to one only, this should be done on the waist or sternum, and not on the foot, considering that the asymmetry in the movements of the feet associated with the initiation of falls will always require the placement bilaterally in any real-life application of the sensor technology. Similarly, in combinations of two to four sensors, they only consider combinations of right and left ankle sensors (that is, never one alone). Finally, Howcroft et al. [60] evaluated the records from the head, pelvis, and both ankles in order to identify the degree of risk of falling. Their results showed that the combination of the records from the head and the posterior pelvis are more reliable than the use of a single sensor. The authors claim that while a single sensor is practical, the best results were obtained with multiple sensors.

In relation to the tests carried out, there were studies that analyzed the accelerations during the performance of validated functional tests, such as the Berg Balance Scale (BBS), Timed Up and Go (TUG) test, Five-Test Sit-To-Stand, Alternate Step test, or the Sensory Organization test (SOT), as done by Shazad et al. [51] and Simila et al. [56]. Their results showed that the use of accelerometers exceeds the sensitivity of the results of the usual functional tests.

The studies that analyzed the gait were performed by conducting walk tests over 15 feet [50], 25 feet [60], and 10 meters [56,61], or the maximum distance reached by the participant walking 5 min on a treadmill [57]. On the contrary, the studies that analyzed the static balance did so through the maintenance of postures in bipodal and monopodal equilibrium, with the eyes open and closed, on the ground and on foam, in tandem position, and as a sole task or simultaneously with carrying out another cognitive task. This battery of conditions resulted, depending on the study analyzed, in a multitude of combinations of tests. To increase the reliability of the records, the authors chose to perform each test for 30 s and to repeat the test battery at least twice [53,58,59].

### 3.3. Testing Environment

All studies took place in a laboratory setting, most likely to allow the use of a gold-standard measurement as a reference (electronic gateway, power platform, camera system with reflective skin markers, etc.), which was the case in six articles [52,53,54,55,61,65].

Aziz et al. [54] asked the participants to simulate seven different types of falls (slipping, stumbling, fainting, changes/incorrect transfer of body weight while sitting, rising from sitting, stretching to reach something, and turning). Their results showed that their accelerometric record is more viable than capturing video (which has cost-related limitations, marker occlusion, and limited measurement volume) for monitoring long-term human movement. However, assessing the risk of falls through its simulation in the laboratory environment, rather than identifying it in a real scenario, is an inappropriate variable that interferes with the generalization of its results.

Similarly, the study by Terrier and Reinard [57] analyzed the gait during the subjects’ performance on a walking belt, which implies the lack of the normal walking conditions encountered in real life.

### 3.4. Data Processing and Evaluated Variables

Those studies that analyzed the accelerations produced during walking [49,50,55,56,57,58,60,61] initially processed the records in the different axes (vertical, anterior-posterior, and mid-lateral) and determined the maximum and minimum values, average, and quadratic mean as an indirect measure of gait disturbances. The average quadratic mean of the three directions (vertical, anterior-posterior, and mid-lateral) was used to calculate the acceleration of the CM. The square root values of the three directions were normalized to the average square root/root mean square ($\mathrm{RMS}=\sqrt{x^{2}+\;y^{2}+\;z^{2}}$), and were represented as a percentage of the average square root.

Subsequently, they analyzed data to extract spatio-temporal variables, such as the length of the step, obtained from the number of steps and the distance travelled, and combined these with the average time in steps (from accelerometer data) to calculate the speed. Heel strikes were identified using the positive spikes of anteroposterior pelvic accelerations and were used to segment each step [66]. The average length of passage can be extracted by calculating the distance travelled against the number of steps taken. According to Hof [67], the spatio-temporal data of the gait was adjusted to the body size, using the length of the legs (LL) as a scale factor. To measure the damping capacity of the accelerations produced from the pelvis to the head, the attenuation coefficient (ATT) in the three axes of the movement was used [68], calculated by the formula: ATT (%) = (1 − RMSH/RMSP) × 100. In addition, it was checked whether there was a harmonic ratio (HR), by the lightness of the walking pattern [69], its rhythm [70], and symmetry of the steps [71], which were calculated following the works proposed by Menz et al. [69]. The higher the HR, the more it will indicate a lighter gait.

On the contrary, when the objective was to analyze the static balance, the records were processed in the different axes (anterior-posterior, mid-lateral, and vertical) and their maximum values, minimum values, average, and root mean square were calculated as an indirect measure of the variability of balance. Subsequently, studies applied linear regression models of the loop [52] of the neuronal network [50], applied linear discrimination [54], and calculated the Euclidean difference [56], various correlation coefficients [59], or the coefficient of Tamimoto [56].

### 3.5. Validation and Reliability Using Gold-Standard Measurements

The GAITRite pressure sensor system (CIR Systems Inc., Easton, PA, USA) was used as a gold-standard reference for gait analysis in two studies [55,61]. This is a reliable instrument for measuring temporal and spatial variables related to gait [72]. The mat has an area of 61 × 366 cm, with 13,824 sensors, separated to 1.27 cm. The measurement is produced by the action of the pressure of the foot when touching the mat. In both studies, there was a significant and high correlation between the electronic gateway and the accelerometric measurements with respect to the speed of travel and cadence. The length and duration of the stroke were significantly and highly correlated.

Aziz et al. [54], used eight cameras to analyze the movement produced in the different types of fall, reproducing at 120 Hz to collect the data of the three spatial dimensions sent by reflective markers located on the skin of the four anatomical positions discussed above. As a gold standard for the evaluation of static equilibrium, Hsieh et al. [53] and Alberts et al. [65] used a force plate (Bertec Inc., Columbus, OH, USA; and NeuroCom Smart Balance Master from NeuroCom International Inc., Clackamas, OR, USA, respectively). Both obtained positive correlation results between the measurements of the force plate and the accelerometric measurements.

The studies of Simila et al. [56] and Shazad et al. [51], in which they compared accelerometric measurements with validated functional clinical tests, used the BBS, which is an instrument that analyses 14 tasks to measure equilibrium performance. Each task is measured from 0 to 4, and the highest score is 56 points. In the studies of Shumway-Cook et al. [73], the authors point out that people who have scores below the value of 49 points have a higher risk of falling, while those who exceed 50 points have a low risk. However, the BBS test consists of a subjective assessment made by a therapist, and, therefore, only evaluates it at a given time, and thus it does not continuously measure the risk of falling during normal life. Both investigations concluded that accelerometry predicts the exact functional balance of the elderly and has the potential to act as a substitute for the BBS.

Another option is the Timed Up and Go (TUG) test, used by Scaglioni-Solano and Aragón-Vargas [58] and Lee et al. [62], which is an excellent indicator of clinically testing the walking ability of patients with musculoskeletal neurological system injury [74,75]. The test involves a person standing, walking for 3 m, making a turn, and again walking back to sit. The time taken by the person to perform the test is closely related to proper maintenance of functional mobility. Those older people who are able to do the test in less than 20 s are considered as independent persons for the tasks that are required to be performed during their daily life, having high values for the BBS test, and can walk at an adequate speed to live in the community (0.5 m/s). Meanwhile, those who spend 30 s or more have a greater dependence in daily activities, need help to walk, and have lower values in the BBS test [75,76]. Scaglioni-Solano and Aragón-Vargas [58] identified that both men and women walked at the same speed (obtaining the same result in the TUG test); however, women walked with a faster cadence and shorter steps than men. This phenomenon has been previously related to anthropometric differences between both sexes (specifically, in relation to size and pelvic width) [77].

Other functional tests employed were the Activities-Specific Balance Confidence scale (ABC) [78] in the study of Howcroft et al. [60] and the Balance Error Scoring System (BESS) in the investigation of Alberts et al. [65]. The ABC scale consists of a questionnaire of 16 questions about confidence in balance to perform specific activities in older adults, and the scores of each of them have integer values between 0 (no confidence) and 100 (total confidence). Total scores are added (range between 0 and 1600) and divided by 16 to obtain the total assessment of each person. Scores below 50 indicate a low level of confidence in balance, values between 50 and 80 indicate a medium level, and values above 80 indicate a high level of confidence. The low values obtained on this scale may limit the activity of the elderly, so that they cannot use the stairs and need auxiliary means to do so [78]. BESS is the most frequently used clinical test to check stable posture in athletes [79]. The test consists of three equilibrium positions with the eyes closed that are held on a firm and foam surface. BESS is considered a valid measurement of postural equilibrium, however, the reliability of the scores obtained between the evaluators and in the evaluator itself has been questioned, since the ratings can reach floor or ceiling effect, which make it limited utility in the clinic.

### 3.6. Application of Technology

Various applications have been recently described for portable systems that include the prevention and early identification of the risk of falls, quantification of the equilibrium level, description of gait, and static balance. The objective of seven studies [49,57,58,60,61,64] was to analyze the progress and its different spatial-temporal parameters characteristics, and discriminate between different types of gait (at different speeds) compared to the results of clinical tests and/or the results of an electronic gateway or motion sensor. In parallel, to analyze the static balance compared to the results of the force platform was the motivation for the study by Saunders et al. [59], and checking if the accelerometric registers were different during different types of fall (fainting, slipping, stumbling, etc.) was the goal of Aziz et al. [54].

The objective of three studies [51,56,62] was to check if the accelerometric measurements outweigh the sensitivity of functional clinical tests, such as the BBS and the TUG test, in all cases, with positive results, and overcoming the discriminative power of patients with and without the risk of falling.

Three other studies [53,63,65] had the objective of evaluating whether the accelerometers included in a smartphone or two different tablet models were valid for the assessment of balance and progress.

Finally, Shin et al. carried out two investigations [52,55] with the objective of discriminating the effects of deficits of vision in the gait.

## 4. Discussion

The aim of this review was to understand the use of accelerometers in order to assess balance in older people. The common themes were fall prevention and early identification of fall risk, balance level classification, and describing gait and static balance.

Until the arrival of accelerometric devices, the reference instrument for the measurement of the balance was the force platform and its determination of the CP of the individual. Comparative studies between the CP and the CM for the evaluation of balance showed the equivalence of both, but with the accelerometer the results are gained quickly and also more cheaply, meaning this is more efficient [43,47].

CP study seems to give a reliable but limited indication of the state of the postural control system since it represents the function of the feet, the last of the intersegmental dynamic system that is the body, while completely ignoring the behavior of more than the whole of the upper half of the body [80,81]. Therefore, the comparison of the reliability and sensitivity of accelerometers to force platforms is, in part, contradictory because its results differ in meaning [69]. This was explained by Panzer et al. [82], who demonstrated in their work that changes in the CP during the evaluation of standing static balance do not always produce changes in the CM, which could indicate different stabilization strategies as it progresses cranially throughout the kinetic chain. In parallel, in another study, it has been shown that accelerometry is more sensitive to the results of different evaluation tests of the equilibrium compared to force platforms [83]. This suggests that posture control may use other equilibrium maintenance strategies that do not fit into the inverted pendulum model of balance [23,82].

This new way of evaluating the balance suggests, in part, that the more conventional view of the biomechanics of postural control is being overcome, which conceived this aptitude as an inverted cone that moved and readapted in space around the articular tibio—peroneo-astragaline axes, and the calcaneocuboid joint (or Chopard joint) [23] that can be represented by the force platforms. Today, on the contrary, the multi-segmental nature of postural control is increasingly accepted [84].

However, the development of this tool in the field of research has not been applied with a consensus on which tests are most appropriate for studying balance, for how long the accelerometric record must be performed, or on what provides more relevant information (equilibrium tests on both feet or just one, with eyes open or closed, on the ground or on an unstable surface, etc.) [69,85].

In relation to the configuration of the devices, this must be less than 55 Hz. This is because the frequencies of human activity are between 0 and 20 Hz, and because 99% of the power signal is contained below 15 Hz [86]. In this way, to capture the full bandwidth of the kinematics, researchers have programmed the sampling of low frequencies.

The placement of the devices has varied between the last vertebrae of the lumbar region and the pelvis. Although the accelerometric data have been collected regularly in the lumbar area to analyze balance, we can find differences in the records obtained due to the different mechanical needs of the area and the internal relations that it maintains with the pelvis. When the accelerometer is placed in the fifth lumbar vertebra, it collects the pressure forces at the base of the sacrum and may be influenced by the rotational movements of the sacrum. Therefore, its placement in the fourth lumbar vertebra is recommended so as not to collect data on the mobility of the pelvic girdle [87].

In relation to the studies that compared the accelerometric records with functional clinical tests, their results showed that the use of accelerometers exceeds the sensitivity of the results of the usual functional tests because of the subjective nature of the evaluation of the examiner, and a minimum variation of four points in the clinical evaluation of BBS is needed to ensure a real change in the functional balance of an older adult [88]. Clinical tests for the evaluation of balance are a widely used tool, but have some limitations, such as the ceiling effect, their subjective nature, and requiring the supervision of an expert, as well as some variation in the administration of the test. In addition, they are not suitable for long-term monitoring (or periodic monitoring), since they require monitoring by the same health professional, which leads to an inefficient use of time and resources. The use of accelerometers overcomes most of these problems.

The studies that analyzed the accelerations during the gait obtained better results, since problems in balance cause changes in the gait [39], while each of the tasks of the BBS evaluates only a certain characteristic, such as coordination or muscular strength. The BBS score is limited to the evaluation of some of the aspects that influence the postural control system, and some other important aspects of balance can be omitted [73].

The sensitivity of the accelerometers for the evaluation of static balance has been tested in numerous investigations [13,89]. The quantification of the accelerations of the CM has demonstrated a discriminatory capacity between different static positions (in balance on one and two feet, with and without vision, and on stable and unstable surfaces [90,91,92]). Data have even been obtained that show greater sensitivity to the accelerometers in front of the force platforms [84]. The accelerometric evaluation of the balance has been compared repeatedly with the results in clinical trials and tests, with positive results in different populations, such as elderly people with a history of falls [93,94] or a cerebrovascular accident [94], children with dyslexia [95], patients with Huntington’s [96], with Parkinson’s disease [47], with progressive cerebellar ataxia [97], and with vestibular disorders [98].

This type of evaluation has a better discriminatory capacity than clinical trials and tests (shown in the previous pages) among the elderly with and without a history of falling, and among elderly and young people [99,100,101]. In addition, simple tests, such as maintenance of the static position on a single foot or tandem, showed reliable values of good to excellent quality [102]. Most studies use the root mean square or RMS (average square root) as a study variable, a measurement of the energy or “force” of a signal (descriptive parameter of the acceleration module on the three axes of space). It is calculated by elevating to the square the instantaneous value of the signal, averaging the squares of the values in time and extracting the square root of the average [103].

Older people show a slower rate of walking compared to younger adults, a phenomenon associated with a reduction in the length of the stroke and, consequently, a decrease in trunk movements (and hence CM) [104]. Through accelerometric analysis, it has also been observed that older people have difficulties adapting their pattern of travel on irregular surfaces [69].

Postural stability along the way can be estimated, on the contrary, from the time in double support, since the subjects with balance problems spend more time with both feet on the floor. However, the subjects with balance problems also walk more slowly and, on the other hand, slow gait is associated with a longer time of permanence in double support [39].

There is a multitude of spacetime parameters, such as speed, length of passage (distance travelled divided by the number of steps detected), step time (duration of the test divided by the number of detected steps), the accelerometer cadence (steps per minute), the regularity or variability of the steps (typical deviation of the time of passage), the coefficient of attenuation of the trunk (percentage of the difference between the vectors of the cranial and pelvic accelerations), and the harmonic relation of the gait (the greater the value, the more regular and symmetrical the steps) [105]. All of these are calculated through different algorithms and statistical techniques [46,106,107].

To date, the description in terms of temporal-spacetime of the body accelerations has shown good results, not only for descriptive purposes, but also for the selection and discrimination between groups with different degrees of athletic conditions, functional and/or cognitive impairment, and/or aging [108], and these results coincide with those expected, considering their vestibular, neuro-muscular physiology and their pathophysiology [109]. On the contrary, the optimal selection of the parameters and their precise definitions are still subject to controversy; so new measures derived from the stochastic dynamic theory have been defined and serve in the same way to describe the patterns of postural control during the gait. Examples of these parameters are the approximate entropy and the control of the entropy, all of them numerical representations of the variability of the different characteristics of the gait [110].

All these measures have been underlined as descriptive parameters of the functioning of the neuro-muscular control system. Thus, with these measures, the degree of functional limitation that implies modification of the gait could be described objectively to reduce the destabilization forces on the CM [111]. Herein, more variability is interpreted as a result of a lower degree of automation of postural control, a lower ability to adapt to changes in the base and/or support surface, and a greater susceptibility to external disturbances [112]. The main results indicate that older people reduce the speed and length of their steps compared to younger adults. The lower speed of the gait is a reflection of the realization of shorter steps and the increase in cadence, characteristics that are related to an increase of the risk of fall. So far, the results of the accelerometric analysis of the gait indicate that the mobility of the trunk and the lower limbs during the gait is reduced, in order to better control movements and maintain stability [105,113].

### Review Limitations

We must be cautious when interpreting the results obtained in this systematic review, since the search had limitations, such as the fact of having done it using eight databases, which was completed with the inclusion of other important works identified in lists and manual searches of references indexed. Another fact to consider in this review are the equations and search terms used within the inclusion criteria, since the use of other terminology would modify the total number of articles found.

However, this review has followed the methodology and criteria used by other similar reviews published recently. For this work, we created a checklist that had as a reference a review of portable technology that analyzed the mobility of the spine [48], as we did not find a standardized instrument in previous works that reflected the quality of the review study.

## 5. Conclusions

The use of accelerometers has the potential to positively influence interventions based on physical exercise to improve balance and prevent falls in older people.

In this review we have found an increase in the use of accelerometers as a measuring instrument for different investigations carried out on gait and balance in adults. However, we have not found a practical application in the daily clinic of accelerometers as an instrument for measuring balance and gait in the consultations.

The most frequently used accelerometer has been the triaxial. The development of accelerometers has been commonly done in an artisanal way, to try to reduce their costs, which has made them insufficiently known by the end users, nor known by the large distributors. Other functions have been added to accelerometers, such as fall prevention and balance analysis, which attempt to know the risk of falls and the motor stability of patients. Long-term monitoring functions have also been described, with the intention of reducing falls, which would allow health professionals to assess the risk of falls in each patient, with the intention of designing more appropriate models for the prevention of falls.

An important quality of accelerometers is the ability to analyze the balance and gait of people in real-life situations, rather than in a laboratory, since this is not a familiar means for patients and can alter the normal gait and balance patterns in the people observed. Some works have compared accelerometers with the gold standard reference, and have found good correlation, reliability, and validity. Although further improvement of the current accelerometers is still necessary, at present, they allow valid and reliable measurements of the CM, which increases its use as a research tool.

Although we have encountered many limitations and difficulties in analyzing the protocols and the different accelerometers used by the authors of the works included in this review, we believe that accelerometers can be valid instruments to analyze different biomechanical aspects in the elderly. Finally, with this review, health professionals and researchers will find the physiological and empirical justification of the methodological criteria and configuration of the accelerometers for the assessment of balance and gait.

## Appendix A

**Table A1:** Quality assessment of included articles.

Quality Index Item	Alberts et al. (2015)	Aziz et al. (2014)	Howcroft et al. (2016)	Hsieh et al. (2019)	Kosse et al. (2015)	Lee et al. (2016)	Park and Woo (2015)
Were the research objectives or aims clearly stated?	2	2	2	2	2	2	2
Was the study design clearly described?	2	0	0	0	0	1	0
Was the study population adequately described?	2	1	2	2	2	2	2
Were the eligibility criteria specified?	2	0	2	2	2	2	0
Was the sampling methodology appropriately described?	0	1	0	0	0	1	0
Was the sample size used justified?	0	0	0	0	0	0	0
Did the method description enable accurate replication?	0	0	1	0	0	0	0
Was the equipment design and set up clearly described?	0	2	0	2	0	0	0
Was accelerometer’s locations accurately described?	1	1	1	0	1	1	2
Was accelerometer’s attachment method clearly described?	2	2	0	1	2	2	2
Was the signal/data handling described?	2	2	2	2	2	2	0
Were the main outcomes measured clearly described?	2	2	2	2	2	2	2
Was the system compared to an acknowledged gold standard?	2	2	2	2	2	2	2
Were measures of reliability/accuracy of the accelerometers used reported?	2	2	2	2	2	2	2
Were the main findings of the study stated?	2	0	0	0	0	0	0
Were the statistical tests appropriate?	1	2	0	2	0	2	2
Were limitations of the study clearly described?	2	0	0	0	0	0	2
Total score (out of 34)	24	19	16	19	17	21	18
Percentage score (%)	70.6	55.9	47.1	55.9	50	61.8	52.9
Quality category	High	Medium	Medium	Medium	Medium	Medium	Medium

**Table A2:** Quality assessment of included articles.

Quality Index Item Number	Saunders et al. (2015)	Scaglioni-Solano and Aragón-Vargas (2015)	Shahzad et al. (2017)	Shin and Yoo (2016)	Shin et al. (2015)	Shin et al. (2016)
Were the research objectives or aims clearly stated?	2	2	2	2	2	2
Was the study design clearly described?	0	0	0	0	0	1
Was the study population adequately described?	2	2	2	2	2	2
Were the eligibility criteria specified?	2	2	2	2	0	2
Was the sampling methodology appropriately described?	0	0	0	0	0	2
Was the sample size used justified?	0	1	0	0	0	0
Did the method description enable accurate replication?	1	0	1	0	1	1
Was the equipment design and set up clearly described?	2	2	2	0	0	2
Was accelerometer’s locations accurately described?	2	2	2	0	2	2
Was accelerometer’s attachment method clearly described?	2	2	1	2	2	2
Was the signal/data handling described?	0	2	0	0	2	2
Were the main outcomes measured clearly described?	2	2	2	0	2	2
Was the system compared to an acknowledged gold standard?	0	2	2	2	2	2
Were measures of reliability/accuracy of the accelerometers used reported?	0	2	2	0	2	0
Were the main findings of the study stated?	0	0	0	0	0	0
Were the statistical tests appropriate?	2	1	1	0	2	2
Were limitations of the study clearly described?	0	0	2	0	2	0
Total score (out of 34)	17	22	21	10	21	24
Percentage score (%)	50	64.7	61.8	29.4	61.8	70.6
Quality category	Medium	Medium	Medium	Medium	Medium	

**Table A3:** Quality assessment of included articles.

Quality Index Item Number	Shin et al. (2018)	Similä et al. (2014)	Terrier and Reynard (2015)	Tung et al. (2014)
Were the research objectives or aims clearly stated?	2	2	2	2
Was the study design clearly described?	1	0	1	1
Was the study population adequately described?	2	2	2	2
Were the eligibility criteria specified?	2	0	1	2
Was the sampling methodology appropriately described?	0	0	0	0
Was the sample size used justified?	0	0	0	0
Did the method description enable accurate replication?	0	0	0	1
Was the equipment design and set up clearly described?	1	0	0	2
Was accelerometer’s locations accurately described?	1	1	2	2
Was accelerometer’s attachment method clearly described?	2	2	2	2
Was the signal/data handling described?	2	2	2	2
Were the main outcomes measured clearly described?	2	2	2	2
Was the system compared to an acknowledged gold standard?	2	2	2	2
Were measures of reliability/accuracy of the accelerometers used reported?	0	2	0	0
Were the main findings of the study stated?	0	0	0	0
Were the statistical tests appropriate?	0	2	2	2
Were limitations of the study clearly described?	2	0	0	0
Total score (out of 34)	19	17	18	22
Percentage score (%)	55.9	50	52.9	64.7
Quality category	Medium	Medium	Medium	Medium

**Table A4:** Methodological details of the research analyzed.

Methodological Item	Alberts et al. (2015)	Aziz et al. (2014)	Howcroft et al. (2016)	Hsieh et al. (2019)
Objective	To determine if an electronic device provides sufficient resolution of the center of gravity movements.	To assess the accuracy of accelerometers to determine the cause of falls.	To identify the best location and combination of accelerometers for status classification of fallers.	To determine if an accelerometer integrated in a smartphone can measure static postural stability and distinguish older adults at high levels of fall risk.
Type of population	Healthy adults	Healthy adults	Older adults with and without previous falls	Older adults
Size sample	49	12	100	30
Mean age of participants (years)	19.5	Unspecified	75.5	65.9
Completed tasks	NeuroCom Sensory Organization Test (SOT)	Falls due to seven causes: slips, trips, fainting and incorrect changes/transfer of body weight while sitting, getting up from sitting, stretching, and turning	Walked 7.62 m	Seven static balance tests along 30 s for each one
Accelerometer used	iPad2 (Apple Inc.)	Microstrain (Inc. G-Link)	Unspecified	Samsung Galaxy S6 (Samsung)
Data acquisition/sampling	Unspecified	128 Hz	Unspecified	Unspecified
Sensor location	Saccrum	Bilaterally in the malleoli, at the waist and the sternum	Head, pelvis, and shanks left and right	Sternum
Data analysis (filters used for signal collection)	Unspecified	Filtered at a low pass using a fourth-order recursive Butterworth filter with a cutoff frequency of 20 Hz	Unspecified	Processed the maximum accelerations on all axes
Comparison with a gold standard	Balance Error Score System (BESS)	8 motion analysis cameras Eagle system (Motion Analysis Corp.) at 120 Hz	Balance Evaluation System test	Force platform (Bertec Inc.)

**Table A5:** Methodological details of the research analyzed.

Methodological Item	Kosse et al. (2015)	Lee et al. (2016)	Park and Woo (2015)	Saunders et al. (2015)
Objective	To establish the validity and reliability of the device for the evaluation of gait and posture.	To classify the participants in fallers and non-fallers according to the accelerations.	To determine the relationship between accelerometry and a foot pressure sensor system to measure gait characteristics.	To evaluate the reliability of the accelerometer.
Type of population	Healthy adults	Older adults	Healthy adults	Older adults
Size sample	60	65	35	20
Mean age of participants (years)	Unspecified	Unspecified	26.3	81
Completed tasks	Walking during 3 minutes	Timed Up and Go Test	Walked 10 m	Four static balance tests along 30 s for each one
Accelerometer used	iPod Touch G4 (Apple Inc.)	Freescale RD3152MMA7260Q (Freescale Semiconductor-NXP)	G-Walk (BTS Bioenginering S.p.A.)	YEI 3-Space Sensor (Yost Engineering Inc.)
Data acquisition/sampling	88–92 Hz	Unspecified	Unspecified	250 Hz
Sensor location	L3	Pelvis, sacrum, and L3 to L5 vertebrae	L5	L3
Data analysis (filters used for signal collection)	Data were interpolated to obtain a constant sampling of 100 Hz	Multi-scale entropy	Unspecified	Applied a filter that eliminated frequencies above 55 Hz
Comparison with a gold standard	Other independent accelerometer	Short Form Berg Balance Scale	GAITRite (CIR Sistem Inc.)	No

**Table A6:** Methodological details of the research analyzed.

Methodological Item	Scaglioni-Solano and Aragón-Vargas (2015)	Shahzad et al. (2017)	Shin and Yoo (2016)	Shin et al. (2015)
Objective	To evaluate gender-related differences in spatial and spatial quality parameters of gait.	To compare the accelerations during gait with the result of the Berg Balance Scale.	To investigate the effects of walking time and trunk acceleration rate.	To investigate the effects of binocular visual acuity on gait velocity and acceleration of the center of mass.
Type of population	Older adults	Older adults	Older women	Older women
Size sample	122	23	25	19
Mean age of participants (years)	69.7	72.9	Unspecified	Unspecified
Completed tasks	Five static balance tests along 30 s for each one	Timed-Up and Go Test, Five-Test Test Sit-to-Stand, and Alternate Step Test	Unspecified	Walked 2 m
Accelerometer used	Technaid SL	Shimmer	Fit Dot Life (Suwon)	Fit Dot Life (Suwon)
Data acquisition/sampling	50 Hz	41 Hz	Unspecified	32 Hz
Sensor location	Head and pelvis	L3–L5	Unspecified	L3
Data analysis (filters used for signal collection)	Fourth-order Butterworth filter (20 Hz cutoff frequency)	Unspecified	Unspecified	Unspecified
Comparison with a gold standard	Timed Up and Go Test	Berg Balance Scale	Unspecified	GAITRite (CIR Sistem Inc.)

**Table A7:** Methodological details of the research analyzed.

Methodological Item	Shin et al. (2016)	Shin et al. (2018)	Similä et al. (2014)	Terrier and Reynard (2015)
Objective	To investigate the effects of square turn and semicircular turn during gait at center of mass.	To explore the effects of binocular visual acuity on the velocity and acceleration of the center of mass.	To investigate the validity of the estimation of the Berg Balance Scale score based on three-dimensional (3D) accelerometry.	To analyze the relationship between age and gait characteristics.
Type of population	Older women	Older women	Neurologic patients and healthy adults	Healthy adults
Size sample	20	26	54	100
Mean age of participants (years)	76.8	Unspecified	Unspecified	44.2
Completed tasks	Walked along a marked path that included two types of turns	Pushed the YBT Kit block (Move2Perform) with one foot in the anterior, posterior-medial, and posterior-lateral directions	Berg Balance Scale	Walked 5 minutes
Accelerometer used	Fit Dot Life (Suwon)	Fit Dot Life (Suwon)	75-Hz alive heart monitor (Alive Technologies)	Physilog Sistem (Gaitup)
Data acquisition/sampling	32 Hz	32 Hz	Unspecified	200 Hz
Sensor location	L3	L3	Lumbar region	5 cm below the sternum
Data analysis (filters used for signal collection)	Unspecified	Unspecified	Five point floating average filter	50 Hz
Comparison with a gold standard	No	No	Berg Balance Scale	No

**Table A8:** Methodological details of the research analyzed.

Methodological Item	Tung et al. (2014)
Objective	To develop and validate a technique to distinguish between five types of walking.
Type of population	Older women with low bone mass
Size sample	18
Mean age of participants (years)	Unspecified
Completed tasks	Walked at five types of gait a distance of 8 m
Accelerometer used	X6-2 Mini (Gulf Coast Data Concepts Inc.)
Data acquisition/sampling	40 Hz
Sensor location	Middle axillary line, at the level of the left iliac crest, and the ankles just above the lateral malleoli
Data analysis (filters used for signal collection)	First, the high pass (0.25 Hz) signals were filtered, then separated into low (0.5–2 Hz) and high (2–10 Hz) frequency bands
Comparison with a gold standard	GAITRite (CIR Sistem Inc.)
